# Integrating validated scores with clinical parameters to predict NIV weaning outcomes: a prospective study in the respiratory ICU

**DOI:** 10.1038/s41598-026-63797-1

**Published:** 2026-07-31

**Authors:** Doaa Gadallah, Hend Mohammed Esmaeel, Hamza Aboalam Mahmoud, Asmaa Ramadan Khalaf

**Affiliations:** 1 Sohag Faculty of Medicine, departement of chest diseases, Sohag University, Sohag, Egypt; 2 Sohag Faculty of Medicine, department of anaesthesia and critical care,Sohag University, Sohag, Egypt

**Keywords:** Non-invasive ventilation, Weaning, HACOR score, APACHE II score, Respiratory ICU, Biomarkers, Diseases, Medical research, Risk factors

## Abstract

Early prediction of successful weaning from non-invasive ventilation (NIV) remains challenging in patients with acute respiratory failure. This study evaluated clinical, laboratory, and severity-score predictors of NIV weaning outcomes among patients admitted to the Respiratory Intensive Care Unit (RICU). This prospective observational cross-sectional study included 150 adult patients with acute respiratory failure, admitted to the RICU and required NIV for > 24 h between April 2024 and March 2025. Clinical characteristics, arterial blood gases, laboratory findings, ventilator settings, and APACHE II score were recorded. HACOR score was assessed after one hour of NIV discontinuation. Patients were classified into successful or failed weaning groups. Successful weaning occurred in 99 patients, while 51 experienced weaning failure. Failure was significantly associated with older age (*p* = 0.001), decompensated cor pulmonale (*p* = 0.003), lower hemoglobin and serum albumin levels (*p* = 0.008 and *p* = 0.005, respectively), higher APACHE II score (18.3 ± 3, *p* < 0.001), and higher HACOR score (8.1 ± 2.1, *p* < 0.001). Previous invasive mechanical ventilation ≥ 2 times was also significantly associated with failure (*p* = 0.001). Multivariate analysis identified age, serum albumin, APACHE II score, HACOR score, and previous invasive ventilation as independent predictors of weaning outcome. Receiver operating characteristic analysis showed good predictive performance for HACOR (AUC 81.6%) and APACHE II (AUC 76.2%) scores (*p* < 0.001 for both). Older age, poor nutritional status, previous invasive ventilation, high APACHE II score, and HACOR score independently predict NIV weaning failure. The incorporating of HACOR score into NIV weaning protocols may improve clinical decision-making and potentially reduce adverse outcomes associated with delayed recognition of respiratory deterioration.

## Introduction

Non-invasive ventilation (NIV) has become a cornerstone in the management of patients with acute or chronic respiratory failure. NIV can be delivered using various ventilatory modes and adjusted according to individual respiratory requirements. It reduces the risks associated with endotracheal intubation and invasive mechanical ventilation (IMV) and improving patient comfort and mobility. Moreover, NIV can be initiated rapidly and discontinued easily when clinical improvement occurs^1^.

Non-invasive positive pressure ventilation (NPPV) has become the first-line ventilatory strategy for specific causes of acute respiratory failure (ARF), including acute exacerbation of chronic obstructive pulmonary disease (COPD) with respiratory acidosis, cardiogenic pulmonary oedema, and severe hypoxemic respiratory failure in immunocompromised patients^2^.

NIV offers a range of advantages like reducing the risk of ventilator-associated pneumonia and avoidance of sedation-related complications, preservation of airway defense mechanisms, and maintenance of swallowing and communication abilities, it also allows patients to eat, drink and communicate^3^.

However, NIV should not delay the endotracheal intubation in patients who fail to respond adequately. Thus, early identification of NIV failure therefore essential to reduce morbidity and mortality^4^. To facilitate early prediction of NIV failure, the researchers developed the HACOR score, which incorporates five variables measured after NIV initiation: Heart rate, Acidosis, Consciousness, Oxygenation, and Respiratory rate. The total score ranges from 0 to 25 points, with higher scores indicating a greater likelihood of NIV failure^5^.

Several studies have reported that many variables can predict weaning outcomes, including disease severity, heart rate, respiratory rate, consciousness, and arterial blood PH. Nevertheless, no single variable has demonstrated sufficient predictive accuracy, and combining multiple clinical parameters may improve predictive accuracy^6,7^.

Previous studies have reported that patients who experience NIV failure, exhibit higher heart rate, lower pH, lower Glasgow Coma Scale (GCS) score, lower oxygenation, and higher respiratory rate than those who are successfully weaned. These variables can be used to predict NIV failure^8,9^.

There is heterogeneity among studies regarding patient populations and clinical settings, and further research is needed to identify independent predictors of NIV weaning outcomes.

Our study aimed to identify clinical, laboratory, and severity-score predictors of NIV weaning outcomes among patients admitted to the Respiratory Intensive Care Unit (RICU).

## Methodology

### Study design, and participants

This prospective observational cross section study was conducted on patients who were admitted to the respiratory ICU in Department of Chest Diseases of Faculty of Medicine, Sohag University and were required non-invasive ventilation for more than 24 h. The study was conducted from April 2024 to March 2025.

The study included adult patients (≥ 18 years) admitted with acute respiratory failure who required NIV for more than 24 h and subsequently met criteria for readiness to wean.

Patients who required invasive mechanical ventilation during the initial management of respiratory failure were excluded.

### Sample size calculation

This study included 150 participants who were received non-invasive ventilation for more than 24 h.

Sample size was calculated using the G*Power statistical program version 3.1.9.4^10^. based on the following assumptions: means ± SDs of HACOR score were 6.7 ± 1.7 and 8.1 ± 2.7 among NIV success and failure groups respectively according to results of a previous study which was conducted among adult patients who were admitted in ICU with respiratory distress and were indicated for NIV in Cairo University Hospital, making an effect size of 2.36^11^. Using α = 0.05, and power = 0.80, the sample size was calculated and yielded at least 46 patients/group. To account for possible dropouts, the sample was increased by 10%, giving a final sample of approximately 50 patients per group.

### Data collection method

All participants underwent detailed history taking from patients or their relatives, including age, sex, occupation, smoking status, comorbidities, and history of previous ICU admissions, clinical Examination and radiological evaluation using chest radiography, high resolution computed tomography scanning of the chest. Baseline laboratory investigations included: Complete blood count (CBC), Liver function tests, Renal function tests, Serum electrolytes. Arterial blood gases analysis was performed at baseline &after one hour of NIV discontinuation: including PH, PaO2, PaCO2, SaO2%, HCO3 measurements.

The APACHE II score (Acute Physiology and Chronic Health Evaluation II) was assessed at baseline, it is a proven tool for assessing baseline illness severity and forecasting hospital mortality risk in critically ill patients. Assessing this severity threshold in the setting of non-invasive ventilation indirectly helps clinicians to identify patients who may be more likely to experience clinical deterioration, which helps choose whether to perform definitive airway treatments. Interpretation of APACHE II: minimum 0 and maximum 71; increasing score is associated with an increasing risk of hospital death^12^.

### NIV protocol

A properly fitted full-face mask was used for NIV delivery. Bi-level positive airway pressure (BiPAP) was selected as the ventilation mode. Initial ventilator settings included: The expiratory positive airway pressure (EPAP) was at 5 cmH2O, and the inspiratory positive airway pressure (IPAP) was at 10 cmH2O.

The pressure settings were subsequently adjusted according to patient tolerance and clinical response. The fractional concentration of oxygen was titrated to maintain the bedside oximeter (SpO2) above 90% and the PaO2 above 60 mmHg.

### Readiness to wean criteria

Patients were considered ready for NIV weaning when the underlying cause of respiratory failure had resolved or significantly improved and all of the following criteria were met: which include arterial pH ≥ 7.35, partial arterial oxygen tension (PaO2) of 60 mm Hg or more, oxygen saturation (SpO2) > 90% on FiO2 ≤ 50%, respiratory rate ≤ 25 / min, heart rate ≤ 120 / min, systolic blood pressure ≥ 90 mmHg and no signs of respiratory distress like agitation, diaphoresis, or anxiety.

### Weaning protocol

NIV support was gradually reduced by decreasing IPAP and EPAP by 2–4 cm H₂O, every 4–6 h while closely monitoring respiratory status. The NIV was discontinued when patients tolerated, IPAP of 6–8 cm of H2O and EPAP of 4–6 cm of H2O.

The patients were assessed after one hour of NIV discontinuation through evaluation of: The arterial blood gas (PH, PaO2, and PaCO2, and SaO2%), HACOR score (PH, PaO2/FiO2, GCS, respiratory rate, and heart rate).

The HACOR score calculation (maximum score of 25) was assessed after one hour of NIV discontinuation as follow:^5^

- HR ≤ 120 beats/minute as 0 point and ≥ 120 beats/minute as 1 point.
- pH ≥ 7.35 as 0 point, 7.30–7.34 as 2 points, 7.25–7.29 as 3 points, and < 7.25 as 4 points.
- GCS 15 as 0 point, 13–14 as 2 points, 11–12 as 5 points, and ≤ 10 as 10 points.
- PaO_2_/FiO_2_ ≥201 as 0 point, 176–200 as 2 points, 151–175 as 3 points, 126–150 as 4 points, and 101–125 as 5 points.
- RR ≤ 30 breaths/minute as 0 point, 31–35 breaths/minute as 1 point, 36–40 breaths/minute as 2 points, 41–45 breaths/minute as 3 points, and ≥ 46 breaths/minute as 4 points.

**Weaning outcomes** were evaluated as weaning success or failure and duration of NIV. Successful weaning of NIV, defined as no requirement of reinstitution of NIV within 48 h of withdrawal^13^. Weaning failure was defined as determination of respiratory failure that required for repeated NIV, intubation, or in the case of patient death within 48 h of NIV weaning^14^.

### Ethics approval and consent to participate

The study protocol was approved by the Medical Research & Ethics Committee of the Faculty of Medicine, Sohag University with Registration number: Soh-Med-22-05-21. All methods were performed in accordance with the relevant guidelines and regulations and performed in accordance with the Declaration of Helsinki. Written Informed consent was obtained from the participants or their legal representatives.

## Results

This study recruited 150 participants who received non- invasive ventilation for acute respiratory failure, successful weaning was achieved in 99 patients (66%), whereas 51 patients (34%) experienced weaning failure. Patients in the successful weaning group had a mean age of 52.2 ± 15 years, significantly younger than in the weaning failure group. Baseline demographic, clinical characteristics and details of underlying chest diseases are presented in Table 1.

Patients with bronchial asthma, COPD and overlap syndrome had significantly higher rate of successful weaning compared with patients with other respiratory diseases (*P* < 0.001).

Decompensated cor pulmonale was significantly associated with weaning failure (*P* = 0.003) (Table 1).

At baseline of vital signs and arterial blood gases, the patients in weaning failure groups had significantly higher respiratory rate with more prevalence of respiratory acidosis, hypercapnia & hypoxemia than in the successful weaning group (*P* < 0.001) as shown in Table 1.

Regarding laboratory investigation, the patients in the weaning failure group had significantly lower level of haemoglobin and serum albumin than in successful weaning groups (*P* = 0.005 & 0.008 for both), no significant differences were detected regarding additional laboratory investigation as shown in Table 2.

The mean APACHE II score was significantly higher in weaning failure group than successful weaning group (18.3 ± 3, *P* < 0.001) (Table 3).

No significant differences were observed between groups regarding initial ventilator settings (IPAP, EPAP, and pressure support). However, a history of previous invasive mechanical ventilation ≥ 2 times (*P* = 0.001) was significantly more prevalent among weaning failure group as shown in Table 3.

At the one-hour follow-up assessment after NIV discontinuation, an increased respiratory rate and elevated systolic blood pressure were significantly associated with weaning failure (*p* = 0.005 and *p* < 0.001, respectively). As regard, ABG parameters did not differ significantly, except for declining of oxygen saturation which was more pronounced in the weaning failure group, as shown in Table 4.

The mean HACOR score after one hour of NIV weaning was significantly higher in weaning failure group than the successful weaning group (8.1 ± 2.1, *P* < 0.001) (Table 4).

The duration of non- invasive ventilation was significantly longer in the weaning failure group (*P* = 0.004) (Table 4).

Univariate analysis identified age, serum albumin level, APACHE II score, HACOR score, duration of NIV, and history of previous invasive mechanical ventilation as significant predictors of weaning outcome (Table 5).

Multivariate regression identified age, albumin, APACHE II, HACOR scores, and previous invasive ventilation as independent predictors of weaning outcome (Table 5).

**Fig. 1:**
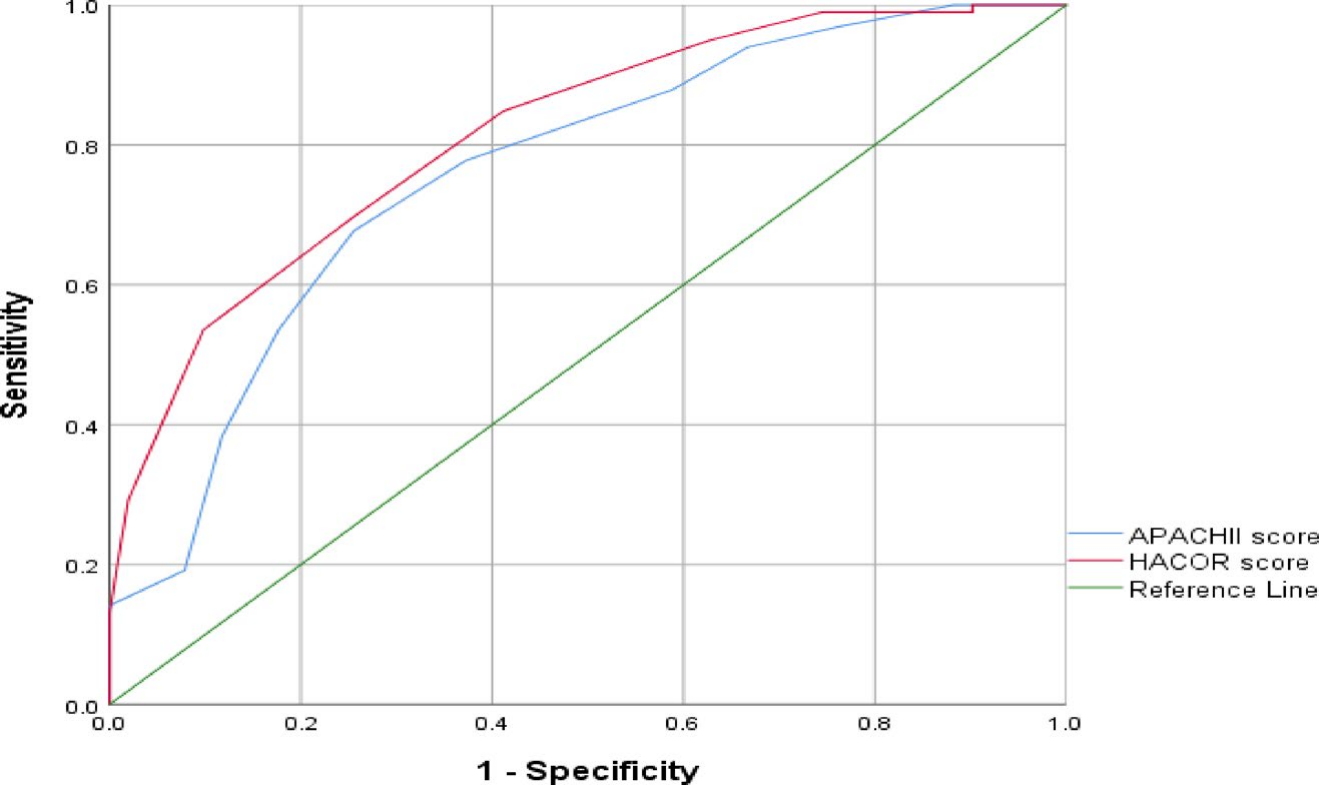
ROC curve of APACHII and HACOR scores in prediction of successful weaning.

The ROC curve analysis confirmed a predictive value of HACOR (AUC 81.6%) and APACHE II scores (AUC 76.2%). The optimal cut off level of HACOR score was 6.5, and APACHE II score was 16.5 (*P* < 0.001 for both) (Table 6 and Fig. 1).

### Statistical analysis

Data was analyzed and presented using Statistical Package for the Social Science (SPSS) version 26 program for data entry and analysis. Descriptive statistics were used to summarize data; in the form of number and percentage for qualitative data, mean ± standard deviation for normally distributed quantitative data in addition to median (IQR) for quantitative not normally distributed data. Shapiro-Wilk test was used to assess normality of data. The association between the studied groups and qualitative data was detected using Chi-squared test, or Fisher’s exact and Monte Carlo significance tests if more than 20% of the total expected cell counts were less than 5. The association between groups and quantitative data was detected by independent sample t test or Mann-Whiteny U test according to normality distribution. Binary logistic regression analysis was performed to detect predictors of successful weaning. Candidate variables were selected a priori based on their clinical relevance and were first evaluated using univariate analysis. Significant Variables in univariate analysis were then included into the multivariable logistic regression model.

Receiver operating characteristics (ROC) curve was used to assess the diagnostic performance of APACHII and HACOR scores in detecting successful weaning. Cutoff values were calculated using Youden’s index. Significance was assessed at p value < 0.05 .

**Table 1:** Comparison between the studied groups as regard baseline clinical data.

Variable	Successful weaning *n* = 99 (66%)	Weaning failure *n* = 51(34%)	*P* value
Age	52.2 ± 15	61 ± 14.2	0.001*^†^
	53 (45:65)	62 (55:68)	
Gender			
Female	51 (51.5)	25 (49)	0.77^‡^
Male	48 (48.5)	26 (51)	
Smoking status			
Non-smoker	55 (55.6)	29 (56.9)	0.99^‡^
Current smoker	16 (16.2)	8 (15.7)	
Ex-smoker	28 (28.3)	14 (27.5)	
Smoking index (*n* = 66)			
Mild-moderate	3 (6.8)	3 (13.6)	0.39^§^
Heavy	41 (93.2)	19 (86.4)	
Acute dyspnea			
No	1 (1)	0	1.0^§^
Yes	98 (99)	51 (100)	
Cough	85 (85.9)	38 (74.5)	0.09^‡^
Chest disease			
Bronchial asthma	8 (8.1)	1 (2)	< 0.001*^ǂ^
COPD	58 (58.6)	18 (35.3)	
Overlap $	11 (11.1)	2 (3.9)	
Bronchiectasis	11 (11.1)	10 (19.6)	
ILD	3 (3)	14 (27.5)	
OHS	8 (8.1)	6 (11.8)	
Comorbidities			
No	63 (63.6)	33 (64.7)	0.11^ǂ^
IHD	21 (21.1)	4 (7.8)	
LC	6 (6.1)	7 (13.7)	
Renal diseases	6 (6.1)	3 (5.9)	
PE	3 (3)	4 (7.8)	
DM	46 (46.5)	23 (45.1)	0.87^‡^
Hypertension	37 (37.4)	17 (33.3)	0.63^‡^
DCP	21 (41.2)	66 (66.7)	0.003*^‡^
Initial vital signs			
RR	29.3 ± 6.6 29 (24:31)	32.6 ± 6.9 32 (27:36)	< 0.001*
SBP	122.5 ± 17	122.6 ± 17.4	0.83^†^
	120 (110:140)	120 (110:130)	
DBP	75.8 ± 10.4	77.4 ± 9.9	0.22^†^
	70 (70:80)	80 (70:80)	
HR	109 ± 16.2	111.4 ± 10.4	0.08^†^
	110 (100:120)	110 (110:120)	
mABP	91.3 ± 12.1 86.7 (83.3:100)	92.5 ± 12 93.3 (83.3:97.5)	0.51^†^
Initial arterial blood gases			
PH	7.34 ± 0.09	7.25 ± 0.05	< 0.001*^¶^
PaCO2	73.9 ± 14.7 72 (64:81)	86.9 ± 13.5 88 (78:95)	< 0.001*^†^
PaO2	56.4 ± 14.6 58 (50:71)	51.9 ± 12.7 50 (42:60)	< 0.001*^†^
SaO2	78.9 ± 14.7 86 (66:92)	70.6 ± 14.4 81 (68:88)	< 0.001*^†^
HCO3	35.9 ± 8.9	34.5 ± 6.4	0.27^¶^

Data is presented in the form of n (%) for qualitative variables and mean ± Sd for quantitative normally distributed data in addition to median (IQR) for quantitative not normally distributed variables, ^†^ Mann-Whiteny U test, ^‡^ Chi squared test, ^§^ fisher’s exact test, ^ǂ^ Monte Carlo simulation of Chi squared test, ^¶^Independent sample t test, *Significant at level < 0.05.

COPD: Chronic obstructive pulmonary disease, ILD; Interstitial lung disease, OHS; Obesity hypoventilation syndrome, IHD; Ischaemic heart disease, LC; liver cirrhosis, PE: pulmonary embolism, DM; Diabetes mellitus, DCP; Decompensated cor pulmonale, RR; Respiratory rate, SBP; Systolic blood pressure, DBP; Diastolic blood pressure, HR; Heart rate, mABP; Mean arterial blood pressure, PaCO2; Partial arterial tension of carbon dioxide, PaO2; Partial arterial tension of oxygen, SaO2; Oxygen saturation, HCO3; Bicarbonate.

**Table 2:** Comparison between the studied groups as regard laboratory investigations.

Variable	Successful weaning (*n* = 99)	Weaning failure (*n* = 51)	*P* value
Na	133.6 ± 6.3 134 (130:138)	134.8 ± 6.4 135 (131:140)	0.39
K	3.9 ± 0.7 3.9 (3.6:4.3)	4.2 ± 0.9 4 (3.5:5)	0.3
Ca	0.9 ± 0.1 0.9 (0.8:0.9)	1.2 ± 1.6 0.9 (0.8:0.9)	0.77
Albumin	3.3 ± 0.3 3.2 (3:3.5)	3 ± 0.7 3 (2.8:3.3)	0.005*
Creatinine	0.9 ± 0.4 0.9 (0.7:1.2)	1 ± 0.6 0.9 (0.6:1.2)	0.73
WBCs	9.9 ± 4.2 8.7 (7:14)	11.1 ± 5 10 (6.8:15)	0.23
Hb	13.4 ± 2.4 13 (12:15)	12.3 ± 2.5 12 (10:15)	0.008*
Platelet	253.1 ± 98.1 253.2 (197:311)	253.5 ± 99.8 240 (200:323)	0.78

Data is presented in the form of mean ± Sd and median (IQR), Significance was detected by Mann-Whiteny U test, * Significant at level < 0.05.

Na; Sodium, K; Potassium, Ca; Calcium, WBCs; White blood cells, HB, Haemoglobin.

**Table 3:** Comparison between the studied groups as regard APACHE II score, and ventilator parameters.

Variable	Successful weaning (*n* = 99)	Weaning failure (*n* = 51)	*P* value
APACHE II score	15.4 ± 2.5	18.3 ± 3	< 0.001*^‡^
IPAP	15.8 ± 3 15 (15:20)	15.2 ± 3.3 15 (15:15)	0.23^†^
EPAP	5 ± 0.3 5 (5:5)	5.2 ± 0.5 5 (5:5)	0.09^†^
PS	10.8 ± 3 10 (10:13)	10 ± 3.5 10 (10:10)	0.15^†^
Previous non-invasive times			
0	78 (78.8)	42 (82.4)	0.45^§^
1	9 (9.1)	6 (11.8)	
≥ 2	12 (12.1)	3 (5.9)	
Previous invasive mechanical ventilation times			
0	88 (88.9)	39 (76.5)	0.001*^ǂ^
1	10 (10.1)	3 (5.9)	
≥ 2	1 (1)	9 (17.6)	

Data is presented in the form of mean ± Sd for normally distributed quantitative data in addition to median (IQR) for quantitative not normally distributed data, ^†^Mann-Whiteny U test, ^‡^ Independent sample t test, ^§^ Chi squared test, ^ǂ^ Monte Carlo simulation of Chi squared test, * Significant at level < 0.05.

IPAP; Inspiratory positive airway pressure, EPAP; Expiratory positive airway pressure, PS; Pressure support.

**Table 4:** Comparison between the studied groups as regard follow up after NIV weaning: vital signs, ABG, and HACOR score.

Variable	Successful weaning (*n* = 99)	Weaning failure (*n* = 51)	*P* value
Vital sings after 1 h			
RR	26.3 ± 6.9	36.9 ± 7.6	0.005*^†^
	25 (22:27.3)	35 (30:40)	
SBP	97.8 ± 11.2	106.4 ± 15.2	< 0.001*^†^
	100 (90:100)	110 (100:115)	
DBP	67.2 ± 6.9	68.8 ± 7.7	0.23^†^
	70 (60:70)	70 (60:70)	
HR	105.7 ± 14.7	111.4 ± 11.1	0.99^†^
	100 (97:120)	110 (110:120)	
mABP	77.5 ± 7.3	81.7 ± 7.7	0.006*^†^
	80 (70:83)	80 (77:87)	
ABG after 1 h			
PH	7.3 ± 0.5	7.3 ± 0.1	0.87^†^
	7.3 (7.27:7.33)	7.3 (7.23:7.4)	
PaO2	68.9 ± 18.4	51 ± 10.3	0.27^†^
	68 (59.5:79.3)	49 (39:60)	
PaCO2	66.8 ± 18.1	71.4 ± 24.2	0.27^†^
	67 (55.8:75.5)	69 (53:92)	
SaO2	89.5 ± 8.3	78.1 ± 13.9	0.57^†^
	92 (87.5:94.3)	80 (66:89)	
HCO3	32.8 ± 7.2	32.8 ± 6.5	0.99^†^
	33 (27:39)	31 (29:40)	
P/F	159.1 ± 82.9	144.7 ± 67.6	0.35^†^
	135 (100:190)	120 (95:183)	
HACOR score after 1 h	5.4 ± 2.1	8.1 ± 2.1	< 0.001*^†^
	5 (4:7)	8 (6:10)	
Duration on non-invasive	7 ± 4.2	10.4 ± 8.1	0.004*^†^
	6 (5:8)	7 (5:11)	
Using steroid	71 (71.7)	31 (60.8)	0.17^§^

Data is presented in the form of mean ± Sd for normally distributed quantitative data in addition to median (IQR) for quantitative not normally distributed data, ^†^Mann-Whiteny U test, ^‡^ Independent sample t test, ^§^ Chi squared test, ^ǂ^ Monte Carlo simulation of Chi squared test, * Significant at level < 0.05.

RR; Respiratory rate, SBP; Systolic blood pressure, DBP; Diastolic blood pressure, HR; Heart rate, mABP; Mean arterial blood pressure, ABG; arterial blood gases PaCO2; Partial arterial tension of carbon dioxide, PaO2; Partial arterial tension of oxygen, SaO2; Oxygen saturation, HCO3; Bicarbonate, P/F; Pao2/Fraction inspired oxygen.

**Table 5:** Logistic regression analysis of predictors of successful weaning.

Variable	Univariate analysis		Multivariate analysis	
	Unadjusted OR (95% CI)	*P* value	Adjusted OR (95% CI)	*P* value
Age	1.04 (1.02:1.07)	0.001*	1.1 (1:1.12)	0.001*
Female gender	1.1 (0.6:2.2)	0.77	–	–
Smoking status				
Non-smoker ^a^	1		–	–
Current smoker	1.1 (0.4:2.8)	0.91		
Ex-smoker	1.1 (0.5:2.3)	0.89		
Comorbidities				
No^a^	1		–	–
IHD	2.8 (0.9:8.7)	0.08		
LC	0.45 (0.1:1.4)	0.18		
Renal	1.05 (0.2:4.5)	0.95		
PE	0.39 (0.1:1.9)	0.24		
DM	1.1 (0.5:2.1)	0.87	–	–
Hypertension	1.2 (0.6:2.4)	0.63	–	–
Albumin	3.3 (1.5:7.3)	0.004*	6.3 (1.8:22.1)	0.004*
APACH II score	0.68 (0.6:0.8)	< 0.001*	0.66 (0.5:0.8)	< 0.001*
HACOR score	0.52 (0.4:0.7)	< 0.001*	0.53 (0.4:0.7)	< 0.001*
IPAP	1.1 (0.95:1.2)	0.26	–	–
EPAP	0.49 (0.2:1.2)	0.11	–	–
PS	1.1 (0.97:1.2)	0.19	–	–
Duration on non-invasive	0.9 (0.8:0.97)	0.004*	0.91 (0.8:1.01)	0.07
Using steroid	1.6 (0.8:3.3)	0.18	–	–
Previous non-invasive times	0.8 (0.3:1.9)	0.61	–	–
Previous invasive mechanical ventilation times	0.41 (0.2:1)	0.05*	0.13 (0.03:0.7)	0.02*

^a^Reference category, * Significant at level < 0.05.

IHD; Ischaemic heart disease, LC; liver cirrhosis, PE: pulmonary embolism, DM; Diabetes mellitus, IPAP; Inspiratory positive airway pressure, EPAP; Expiratory positive airway pressure, PS; Pressure support.

**Table 6:** Diagnostic performance of APACHII and HACOR scores in prediction of successful weaning.

Variable	AUC	95% CI	*P* value	Cutoff point	Sensitivity	Specificity
APACHII	76.2%	0.7:0.8	< 0.001*	16.5	67.7%	74.5%
HACOR	81.6%	0.7:0.9	< 0.001*	6.5	69.7%	74.5%

## Discussion

NIV has gained acceptance worldwide over the past decade and is currently considered the first line for the ventilatory support modality for patients with ARF especially those related to exacerbation of obstructive airway disease and acute decompensated heart failure. It is now commonly used in the emergency department (ED). Many studies have shown that the early initiation of NIV is strongly encouraged in the ED for these patients, it reduces morbidity and mortality and when used appropriately, it can even shorten hospital stay^15^. However, determining the optimal method of evaluating weaning from NIV, remains challenging in patients with acute respiratory failure. Therefore, in the present study, we evaluated the clinical, laboratory, and severity-score predictors of NIV weaning outcomes among patients admitted to the Respiratory Intensive Care Unit (RICU).

In the present study, two thirds of patients (66%) were successfully weaned from NIV, whereas one third (34%) experienced weaning failure, this result was matched with Kattinanon N, et al., who reported that (58.9%) patients had successful weaning from NIPPV, while 203 (41.1%) patients had weaning failure, The observed differences in weaning outcomes across studies may be attributed to variations in disease severity and patients’ characteristics^16^.

Patients who experienced weaning failure were significantly older; this result disagreed with the study of Yu J et al., who found that there were no significant differences were detected between the successful weaning and weaning failure in terms of gender, and age^17^. Our results may be explained by the age-related decline in respiratory mechanics, muscle strength, and comorbidity burden negatively impacts the ability to sustain spontaneous ventilation^18^.

The distribution of primary chest diseases showed a highly significant difference between the study groups. COPD predominated among patients who were successfully weaned, this result matched the study of Yu J et al., who evaluated NIV in patients with acute exacerbations of COPD (AECOPD) and acute respiratory failure and reported similar success rates^17^. Likewise Colaianni-Alfonso N et al., demonstrated that NIV resulted in greater clinical improvement than high-flow nasal cannula therapy in COPD patients with acute hypercapnic respiratory failure^19^, while Tan D et al., study reported that the patients who used of HFNC after extubation experienced better tolerance and comfort than the use of NIV^20^. Conversely, patients with interstitial lung disease (ILD) and bronchiectasis were more prevalent among weaning failure group. Restrictive pathologies such as ILD reduce lung compliance and gas exchange capacity, making liberation from ventilatory support more challenging^21^.

The association between initial tachypnoea, acidosis, hypercapnia, and NIV weaning failure in this study is consistent with the findings of Kattinanon N, et al., study who found that there was significant association between weaning failure group and increasing of respiratory rate (*P* = 0.002) and hypercapnia (*P* = 0.037)^16^. Therefore, respiratory rate may be considered a predictor of NIV discontinuation outcomes, as it increases with disease severity and is associated with both weaning failure and the need for endotracheal intubation^8^.

Low serum albumin level was significantly associated with weaning failure in the present work. This result agrees with the study of Wafy et al., who reported that serum albumin was higher among successful weaning group^22^, and similar results were reported by Wang et al.^23^. Serum albumin is an important marker in the clinical assessment of nutritional status of patients, and hypoalbuminemia is considered as an indicator of malnutrition and systemic inflammatory response syndrome^24^. Reduced serum albumin levels may impair immune function, weaken respiratory muscles, decrease ventilatory capacity, and reduce pulmonary function. Consequently, low albumin levels may adversely affect the effectiveness of NIV and increase the likelihood of prolonged or failed weaning^25^.

In this study, patients in the weaning failure group had significantly lower haemoglobin than those in successful weaning groups. This is in agreement with the study of Leela-Amornsin S, et al., who identified that there was a significant difference in the haemoglobin level between the two weaning outcome groups^26^, This association may be explained by the critical role of haemoglobin in oxygen delivery, as reduced haemoglobin levels can adversely affect cardiac workload, increase the work of breathing, and impair respiratory muscle endurance^27^.

APACHE II score was significantly higher in the weaning failure group than the successful weaning group. This finding is consistent with the study by Sun W. et al. who found that Patients with failed NIV had significantly higher APACHE-II score compared with those who were successfully weaned (19.78 ± 4.09 vs. 13.77 ± 3.68; *P* < 0.001)^28^.

Regarding the initial Ventilator setting (IPAP, EPAP, PS) there was no significant differences between the successful and failure weaning groups. In line with this, the study of Leela-Amornsin S, et al. study reported that there were no statistical differences in IPAP and EPAP (*P* > 0.05) between the success and failure weaning groups, indicating that pressure settings alone do not adequately explain weaning failure^26^.

Notably, we find that the history of previous invasive mechanical ventilation ≥ 2 times was significantly more prevalent among weaning failure. Repeated intubation can lead to diaphragmatic dysfunction and prolonged respiratory muscle weakness thereby, increasing dependence on ventilator support. The concept of cumulative Ventilator-Induced Diaphragmatic Dysfunction (VIDD), which has been extensively discussed in the literature, is extremely congruent with this finding.

The duration of non-invasive ventilation was significantly longer in the weaning failure group. Yu J et al., similarly reported a significant differences in NIV duration between the successful and failed weaning groups (5.0 vs. 13.0 days; *p* < 0.001)^17^.

The one-hour follow-up assessment after NIV weaning revealed an increased respiratory rate, elevated systolic blood pressure, and a decline in oxygen saturation among patients who experienced weaning failure. These findings indicate an inability to maintain adequate spontaneous ventilation, likely resulting from an increased work of breathing and respiratory muscle fatigue.

Nonetheless, there were no significant differences between the two groups in any of the weaning indices or ABG parameters, these findings suggest that clinical signs may be more sensitive than laboratory values during the early assessment of weaning outcomes.

Regarding the evaluation of HACOR scores one hour after NIV discontinuation, the score was significantly higher in the weaning failure group. These findings similar to those reported by Chaudhuri S et al., study of the utility of the One-time HACOR Score as a predictor of weaning failure from mechanical ventilation. They reported longer median and interquartile range (IQR) of the HACOR score in the successful weaning group; (0–3) than the failed weaning group; 6 (5–8) (*p*-value < 0.001)^29^. Likewise findings of Teh YH et al. were consistent with these results^30^.

we selected the 1-hour post-discontinuation period as our primary goal, in order to maximize clinical utility by facilitating the early recognition of respiratory exhaustion and reducing the well-established mortality risks associated with delayed respiratory re-intervention.

ROC curve revealed that the HACOR score maintains significant discriminative performance after one hour of NIV discontinuation (AUC 81.6) with the optimal cutoff level 6.5, This cutoff may enable physicians to identify patients at high risk of weaning failure and intervene promptly before the development of severe respiratory deterioration. The score demonstrated a satisfactory diagnostic performance pattern with sensitivity 69.7% and specificity 74.5% with P value < 0.001, this result matched with the study of Chaudhuri S et al., who found that HACOR score ≥ 5 predicted NIV weaning failure with sensitivity 83.8%, specificity 96.4%, area under the curve (AUC) 0.950, and 95% confidence interval (CI) [0.907–0.993], *p* < 0.001^29^.

ROC curve analysis also confirmed the predictive value of APACHE II scores (AUC 76.2%), with the optimal cut-off value 16.5. This result is in agreement with the study of Sun W et al. who demonstrated that APACHE II scores (> 16.5) independently associated with NIV failure and mortality among patients with pneumonia-induced ARDS, with a sensitivity of 84.4%, specificity of 68.7%, diagnostic accuracy of 76.3%, and an AUC of 0.818 (95% CI 0.745–0.891)^28^.

Multivariate analysis in the current study identified age, albumin, APACHE II, HACOR scores, and previous invasive ventilation as independent predictors of weaning outcomes. These data suggest that age, nutritional status, and indices of disease severity, including the HACOR and APACHE II scores, play pivotal roles in determining the likelihood of achieving ventilatory independence. The Duration of NIV use showed borderline significance.

### Limitations and strengths of the study

This study has several limitations that should be acknowledged. First, it was conducted at a single center, which may limit the generalizability of the findings to other institutions with different patient populations, clinical practices, or NIV weaning protocols. Second, although the sample size was adequate for identifying major predictors, a larger cohort would have provided greater statistical power and allowed more detailed subgroup analyses, particularly across different aetiologies of respiratory failure. Third, the heterogeneity of underlying respiratory diagnoses may have introduced confounding effects, as some conditions inherently respond better to NIV than others. Fourth, the study focused primarily on short-term weaning outcomes and did not evaluate long-term endpoints such as mortality, re-intubation rates, or hospital readmissions. These limitations can be addressed in future research.

On the other hand this work encompasses several distinctive methodological stregnths. First the prospective design with measurements at predefined points of baseline and one hour follow up after NIV discontinuation, increases internal validity. Second, comprehensive data collection: clinical exam, gasometric data, labs, ventilator settings, and validated severity scores. In addition, pragmatic NIV protocol is prescribed.

Third, multivariable analysis identifies independent predictors rather than relying on univariate associations. Fourth, it provides actionable threshold through reporting optimal cutoff for HACOR and APACHEII scores. Fifth, the study assessed clinically relevant outcomes (weaning success versus failure).

## Conclusion

Successful weaning from non-invasive ventilation (NIV) remains a major challenge in patients with acute respiratory failure. In this prospective study, older age, hypoalbuminemia, higher APACHE II and HACOR scores, and a history of previous invasive mechanical ventilation emerged as independent predictors of NIV weaning failure. Furthermore, the clinical application of an early, objective assessment at 60 min after NIV discontinuation demonstrated significant predictive value and may facilitate the early identification of patients at high risk of weaning failure, enabling timely respiratory re-intervention.

Incorporating the HACOR score into NIV weaning protocols may improve clinical decision-making and potentially reduce adverse outcomes associated with delayed recognition of respiratory deterioration. Further multicenter studies with larger sample sizes are warranted to validate these findings and establish standardized NIV weaning protocols.

## Data Availability

All data generated or analyzed during this study are included in this published article.

## References

[1] Criner, G. J. et al. Clinical review of non-invasive ventilation. *Eur. Respir J.* **64** (5), 2400396. 10.1183/13993003.00396-2024 (2024). 39227076 10.1183/13993003.00396-2024 PMC11540995

[2] Rochwerg, B. et al. Members Of The Task Force. Official ERS/ATS clinical practice guidelines: noninvasive ventilation for acute respiratory failure. *Eur. Respir J.* **50** (2), 1602426. 10.1183/13993003.02426-2016 (2017). 28860265 10.1183/13993003.02426-2016

[3] Nava, S. & Hill, N. Non-invasive ventilation in acute respiratory failure. *Lancet* **374** (9685), 250–259. 10.1016/S0140-6736(09)60496-7 (2009). 19616722 10.1016/S0140-6736(09)60496-7 PMC7138083

[4] Mosier, J. M. et al. Failed noninvasive positive-pressure ventilation is associated with an increased risk of intubation-related complications. *Ann. Intensive Care*. **5**, 4. 10.1186/s13613-015-0044-1 (2015). 25852964 10.1186/s13613-015-0044-1 PMC4385202

[5] Duan, J., Han, X., Bai, L., Zhou, L. & Huang, S. Assessment of heart rate, acidosis, consciousness, oxygenation, and respiratory rate to predict noninvasive ventilation failure in hypoxemic patients. *Intensive Care Med.* **43** (2), 192–199. 10.1007/s00134-016-4601-3 (2017). 27812731 10.1007/s00134-016-4601-3

[6] Ko, B. S. et al. Early failure of noninvasive ventilation in chronic obstructive pulmonary disease with acute hypercapnic respiratory failure. *Intern. Emerg. Med.* **10** (7), 855–860. 10.1007/s11739-015-1293-6 (2015). 26341216 10.1007/s11739-015-1293-6

[7] Fiorino, S. et al. Efficacy of non-invasive mechanical ventilation in the general ward in patients with chronic obstructive pulmonary disease admitted for hypercapnic acute respiratory failure and pH < 7.35: a feasibility pilot study. *Intern. Med. J.* **45** (5), 527–537. 10.1111/imj.12726 (2015). 25684643 10.1111/imj.12726

[8] Yoshida, Y. et al. Factors predicting successful noninvasive ventilation in acute lung injury. *J. Anesth.* **22** (3), 201–206. 10.1007/s00540-008-0637-z (2008). 18685924 10.1007/s00540-008-0637-z

[9] Nicolini, A. et al. Early non-invasive ventilation treatment for respiratory failure due to severe community-acquired pneumonia. *Clin. Respir J.* **10** (1), 98–103. 10.1111/crj.12184 (2016). 25043135 10.1111/crj.12184

[10] Faul, F., Erdfelder, E., Lang, A. G. & Buchner, A. G*Power 3: a flexible statistical power analysis program for the social, behavioral, and biomedical sciences. *Behav. Res. Methods*. **39** (2), 175–191. 10.3758/bf03193146 (2007). 17695343 10.3758/bf03193146

[11] Ahmed, W. M. K., Samir, M., Andraos, A. W. & Hosny, M. Radiological and clinical perspectives to predict failure of noninvasive ventilation in acute respiratory failure. *Egypt. J. Crit. Care Med.* **10**, 1–6. 10.1097/ej9.0000000000000047 (2023).

[12] Knaus, W. A., Draper, E. A., Wagner, D. P. & Zimmerman, J. E. APACHE II: a severity of disease classification system. *Crit. Care Med.* **13** (10), 818–829 (1985). 3928249

[13] Venkatnarayan, K. et al. A comparison of three strategies for withdrawal of noninvasive ventilation in chronic obstructive pulmonary disease with acute respiratory failure: Randomized trial. *Lung India*. **37** (1), 3–7. 10.4103/lungindia.lungindia_335_19 (2020). 31898613 10.4103/lungindia.lungindia_335_19 PMC6961096

[14] Boles, J. M. et al. Weaning from mechanical ventilation. *Eur. Respir J.* **29** (5), 1033–1056. 10.1183/09031936.00010206 (2007). 17470624 10.1183/09031936.00010206

[15] Hill, N. S. Where should noninvasive ventilation be delivered? *Respir Care*. **54** (1), 62–70 (2009). 19111107

[16] Kattinanon, N. et al. A Clinical Score for Predicting Successful Weaning from Noninvasive Positive Pressure Ventilation in Emergency Department; a Retrospective Cohort Study. *Arch. Acad. Emerg. Med.* **12** (1), e15. 10.22037/aaem.v12i1.2173 (2024). 38371444 10.22037/aaem.v12i1.2173 PMC10871050

[17] Yu, J., Lee, M. R., Chen, C. T., Lin, Y. T. & How, C. K. Predictors of Successful Weaning from Noninvasive Ventilation in Patients with Acute Exacerbation of Chronic Obstructive Pulmonary Disease: A Single-Center Retrospective Cohort Study. *Lung* **199** (5), 457–466. 10.1007/s00408-021-00469-z (2021). Epub 2021 Aug 21. 34420091 10.1007/s00408-021-00469-z PMC8380010

[18] Sharma, G. & Goodwin, J. Effect of aging on respiratory system physiology and immunology. *Clin. Interv Aging*. **1** (3), 253–260. 10.2147/ciia.2006.1.3.253 (2006). 18046878 10.2147/ciia.2006.1.3.253 PMC2695176

[19] Colaianni-Alfonso, N. et al. High-flow nasal cannula versus non-invasive ventilation for acute exacerbations of chronic obstructive pulmonary disease with acute-moderate hypercapnic respiratory failure: a retrospective study. *Front. Med. (Lausanne)*. **12**, 1582749. 10.3389/fmed.2025.1582749 (2025). 40606468 10.3389/fmed.2025.1582749 PMC12213871

[20] Tan, D. et al. High-flow nasal cannula oxygen therapy versus non-invasive ventilation for chronic obstructive pulmonary disease patients after extubation: a multicenter, randomized controlled trial. *Crit. Care*. **24** (1), 489. 10.1186/s13054-020-03214-9 (2020). 32762701 10.1186/s13054-020-03214-9 PMC7407427

[21] Aliberti, S. et al. Non-invasive mechanical ventilation in patients with diffuse interstitial lung diseases. *BMC Pulm Med.* **14**, 194. 10.1186/1471-2466-14-19 (2014). 25476922 10.1186/1471-2466-14-194 PMC4269964

[22] Wafy, S. M., Bayomy, H. A., Mohamed, S. S. & Ahmed, M. K. Predictors of Non-Invasive Ventilation Failure in Respiratory Intensive Care. *Egypt. J. Crit. Care Med.* **8**, 118–123 (2021).

[23] Wang, J. et al. Influencing factors of noninvasive positive pressure ventilation in the treatment of respiratory failure: a 10-year study in one single center. *Eur. J. Med. Res.* **26** (1), 136. 10.1186/s40001-021-00615-6 (2021). 34861893 10.1186/s40001-021-00615-6 PMC8641230

[24] Akirov, A., Masri-Iraqi, H., Atamna, A. & Shimon, I. Low Albumin Levels Are Associated with Mortality Risk in Hospitalized Patients. Am J Med. ;130(12):1465.e11-1465.e19. (2017). 10.1016/j.amjmed.2017.07.020. Epub 2017 Aug 9. Erratum in: Am J Med. 2020;133(5):646. doi: 10.1016/j.amjmed.2020.02.001. 10.1016/j.amjmed.2017.07.020 28803138

[25] Soeters, P. B., Wolfe, R. R. & Shenkin, A. Hypoalbuminemia: Pathogenesis and Clinical Significance. *JPEN J. Parenter. Enter. Nutr.* **43** (2), 181–193. 10.1002/jpen.1451 (2019). 10.1002/jpen.1451 PMC7379941 30288759

[26] Leela-Amornsin, S., Triganjananun, C., Yuksen, C., Jenpanitpong, C. & Watcharakitpaisan, S. Clinical Prediction Score for Successful Weaning from Noninvasive Positive Pressure Ventilation (NIPPV) in Emergency Department; a Retrospective Cohort Study. *Arch. Acad. Emerg. Med.* **10** (1), e79. 10.22037/aaem.v10i1.1769 (2022). 36426167 10.22037/aaem.v10i1.1769 PMC9676697

[27] Ouellette, D. R. The impact of anemia in patients with respiratory failure. *Chest* **128** (5 Suppl 2), 576S–582S. 10.1378/chest.128.5_suppl_2.576S (2005). 16306056 10.1378/chest.128.5_suppl_2.576S

[28] Sun, W. et al. A combination of the APACHE II score, neutrophil/lymphocyte ratio, and expired tidal volume could predict non-invasive ventilation failure in pneumonia-induced mild to moderate acute respiratory distress syndrome patients. *Ann. Transl Med.* **10** (7), 407. 10.21037/atm-22-536 (2022). 35530968 10.21037/atm-22-536 PMC9073780

[29] Chaudhuri, S. et al. Utility of the One-time HACOR Score as a Predictor of Weaning Failure from Mechanical Ventilation: A Prospective Observational Study. *Indian J. Crit. Care Med.* **26** (8), 900–905. 10.5005/jp-journals-10071-24280 (2022). 36042760 10.5005/jp-journals-10071-24280 PMC9363817

[30] Teh, Y. H., Nazri, M. Z. A. M., Azhar, A. M. N. & Alip, R. M. HACOR Score in Predicting Non-invasive Ventilation Failure in Acute Decompensated Heart Failure and AECOAD Patients. *Eurasian J. Emerg. Med.* **21** (3), 165–175. 10.4274/eajem.galenos.2022.09734 (2022).

